# Sterility in the Male

**Published:** 1888-04

**Authors:** 


### *STERILITY IN THE MALE.*

Dr. Belfield emphasizes the fact, largely ignored in practice, that potence does not secure fecundity. Natural desire, complete erection, copious and well-timed ejaculation, and intense orgasm, may all be exhibited by an absolutely sterile man. The responsibility for a childless marriage is popularly, and but too often professionally, attributed to the wife; investigation of the husband is omitted, or limited to ascertaining that the act is normally performed. The wife is treated; intra-uterine applications are made, pessaries applied, the cervical canal enlarged; yet no conception takes place, because no normal spermatozoa are deposited.

In every case in which medical advice is sought as to barrenness in marriage, the first examination should be directed to the semen, no matter how vigorous and potent the husband may be. Sterility without impotence may be due to the absence of normal spermatozoa from the semen—azoospermism ; or to the failure to ejaculate—aspermatism. The most frequent cause of aspermatism is urethral stricture; a contraction which may offer no serious obstruction to urination, may from the compression during erection prevent the passage of semen. Gross suggested that the occlusion is produced wholly or partly by spasmodic contraction of the urethra at the sensitive point. A contracted meatus or tight phimosis in the same way might prevent the discharge. Other causes are congenital defects or malformations, inflammatory occlusions, concretions formed in the seminal vesicles or prostate, etc.

Azoospermism is the most frequent cause of male sterility, and is by no means rare. This cause was assigned by Kehrer to fourteen out of forty childless marriages. The most frequent causes are bilateral obliteration of the epididymis and vas deferens; bilateral orchitis; arrest of growth of the testicles—the latter common in cryptorchids. Of eighty-three cases of double gonorrhæal epididymitis, seventy-six were afterwards without sperm-cells in the semen. The facts cited show that childless marriages are often referable to the male.—*Indiana Med. Journal.*

